# Error-Related Cognitive Control and Behavioral Adaptation Mechanisms in the Context of Motor Functioning and Anxiety

**DOI:** 10.3389/fnhum.2021.615616

**Published:** 2021-02-05

**Authors:** Marta Topor, Bertram Opitz, Hayley C. Leonard

**Affiliations:** School of Psychology, University of Surrey, Guildford, United Kingdom

**Keywords:** motor skills, cognitive control, behavioral adaptation, anxiety, error-related negativity, post-error slowing

## Abstract

Motor proficiency reflects the ability to perform precise and coordinated movements in different contexts. Previous research suggests that different profiles of motor proficiency may be associated with different cognitive functioning characteristics thus suggesting an interaction between cognitive and motor processes. The current study investigated this interaction in the general population of healthy adults with different profiles of motor proficiency by focusing on error-related cognitive control and behavioral adaptation mechanisms. In addition, the impact of these processes was assessed in terms of trait anxiety and worries. Forty healthy adults were divided into high and low motor proficiency groups based on an assessment of their motor skills. Using electroencephalography during a flanker task, error-related negativity (ERN) was measured as the neural indicator of cognitive control. Post-error slowing (PES) was measured to represent behavioral adaptation. Participants also completed an anxiety assessment questionnaire. Participants in the high motor proficiency group achieved better task accuracy and showed relatively enhanced cognitive control through increased ERN. Contrastingly, individuals in the lower motor proficiency group achieved poorer accuracy whilst showing some evidence of compensation through increased PES. Trait anxiety reflecting general worries was found to be correlated with motor functioning, but the study could not provide evidence that this was related to cognitive or behavioral control mechanisms. The interaction between cognitive and motor processes observed in this study is unique for healthy and sub-clinical populations and provides a baseline for the interpretation of similar investigations in individuals with motor disorders.

## Introduction

The relationship between motor and cognitive functioning (from now referred to as the M-C interaction) is an important topic explored through different research approaches. It has been studied specifically in the context of child development (van der Fels et al., 2015) and health conditions affecting movement including, amongst others, neurodevelopmental conditions such as developmental coordination disorder (DCD; Sartori et al., 2020), neurodegenerative conditions such as Parkinson's disease (PD; Sollinger et al., 2010), as well as stroke (Plummer et al., 2013), and acquired brain injury (Damiano et al., 2016). Research concerning the M-C interaction in the adult general population is rare but has recently gained more attention with the aim of investigating how executive and cognitive processes may be associated with different profiles of motor ability and proficiency (Marchetti et al., 2015; Stuhr et al., 2018; Ludyga et al., 2019).

### Observations of the Motor-Cognitive Interaction

Motor proficiency is the measure of an individual's ability to perform intended motor actions. These may include fine and gross motor skills, assessed based on precision, agility, and coordination (Morley et al., 2015). It has been reported that individuals with different profiles of motor proficiency also present with different cognitive ability profiles (Piek et al., 2008) thus suggesting a modulation in the M-C interaction. However, the nature of this phenomenon is not yet well-understood.

The latest advancements in the attempt to understand the M-C interaction in the general population with different motor proficiency profiles suggest that this interaction may link specific faculties of executive functioning with different motor domains (Marchetti et al., 2015; Ludyga et al., 2019). For instance, working memory may be associated with fine motor skills (Stuhr et al., 2018) as well as locomotor skills (Ludyga et al., 2019), whilst inhibitory control may be associated with balance (Rigoli et al., 2012; Stuhr et al., 2018). A common suggestion in this line of research is that specific cognitive processes may help to facilitate control over an individual's action in order to improve task performance. For instance, Stuhr et al. (2018) argue that the M-C interaction is mediated by task difficulty, and cognitive control processes are engaged to facilitate successful completion of tasks that are difficult or novel. Marchetti et al. (2015), on the other hand, explore the possibility that the engagement of cognitive processes such as inhibition may facilitate good performance on motor tasks that are challenging and require a strategic approach. Thus, the M-C interaction in the context of motor proficiency can be viewed to be goal-directed, helping individuals to perform to the best of their ability despite the challenges of the tasks and activities being undertaken (Marchetti et al., 2015; Stuhr et al., 2018).

### Control Processes on the Neural Level

The M-C interaction can be explored further at the neural level. For instance, motor actions have been reported to have a physiological link with working memory, semantic processing, and language as evident through investigations using electroencephalography (EEG; Amsel et al., 2013; Spiegel et al., 2013; Koester and Schack, 2016; Gunduz Can et al., 2017). From this perspective, it can be understood that high motor demand may lead to a physiological reduction in cognitive resources and thus impact cognitive performance. To the best of our knowledge, the M-C interaction on the neural level with the focus on motor proficiency has only been studied by observing the presentation and performance of individuals with motor disorders, e.g., DCD. In DCD, individuals experience significant difficulties with the execution of coordinated movements but also problems with executive functions (Leonard et al., 2015; Sartori et al., 2020). Querne et al. (2008) conducted a study using functional magnetic resonance imagery (fMRI) with children completing a standard Go/NoGo paradigm, in which responses must be provided or inhibited depending on the instructions. Children with DCD achieved similar accuracy to healthy children on the task, despite slower responses. The fMRI results suggest that children with DCD may have a stronger activation of the anterior cingulate cortex (ACC), which may help to maintain good accuracy on the task whilst compensating for motor difficulties.

The ACC is a key structure within the fronto-striatal network. It is commonly regarded as a key structure for cognitive and emotional control (Bush et al., 2000). It has been reported to facilitate emotion regulation (Stevens et al., 2011), to aid episodic memory formation by providing reward—related information to the hippocampal memory system (Rolls, 2019), and gets activated with high working memory load to signal the need for control (Gray and Braver, 2002). The ACC is also an integral part of the Human Error Processing System (Holroyd and Coles, 2002) exhibiting control over one's actions for successful task performance. A recent update on the model acknowledges a wider role of the ACC in the updating of goals, available actions and their outcomes, as well as the selection and the execution of appropriate actions (Brown and Alexander, 2017). Thus, the ACC is not only involved in cognitive control but is also relevant for motor functioning. The ACC has been shown to modulate the activity in the supplementary motor area during proactive and reactive motor tasks (Asemi et al., 2015) and facilitates motor coordination during voluntary movements (Wenderoth et al., 2005).

A vital element of performance control in the ACC is the prediction error, which compares the expected to actual outcomes and reflects positive or negative surprise. The ACC uses the prediction error signal to evaluate the available actions and navigate the selection of appropriate actions in the future (Robbins, 2009). Prediction errors can be measured in the form of the error-related negativity (ERN), a response-locked component of the event-related potential observed with electroencephalography following error commission. It occurs about 50 ms post erroneous response and larger negative amplitudes indicate stronger engagement of the ACC (Shenhav et al., 2013; Hauser et al., 2014).

ERN is commonly studied in clinical populations, both neurodevelopmental and neurodegenerative. For instance, it has been reported that patients with PD have generally lower ERN compared to a healthy control group suggesting that error sensitivity may be affected by PD-related alterations in the dopaminergic systems (Stemmer et al., 2007). Similarly, children with attention deficit hyperactivity disorder (ADHD), which is also known for alterations in dopaminergic availability, showed attenuated ERN and error evaluation (Rosch and Hawk, 2013). ERN was recently investigated in Tourette's syndrome (TS), a neurodevelopmental motor disorder, which is characterized by difficulties with the control of involuntary movements (Bloch and Leckman, 2009). Schüller et al. (2018) showed that adult participants with TS could perform the stop-signal task, which requires the inhibition of responses when instructed to do so, with accuracy similar to that of healthy participants. However, their ERN amplitude was significantly larger. This pattern can be compared to the results observed using fMRI in children with DCD by Querne et al. (2008). Both studies suggest that participants with motor disorders perform cognitive tasks (Go/NoGo or stop-signal) with the same accuracy as their peers with no motor conditions, but they present with stronger neural activation. These enhanced cognitive processes may facilitate task performance of individuals with motor disorders as a way of compensation for their motor control difficulties.

### Control Processes at the Behavioral Level

Performance control processes can also be studied with regards to the changes in an individual's behavior to achieve better accuracy and performance. This occurs when the ACC detects a discrepancy between the expected and actual outcomes of an action (Brown and Alexander, 2017). One measure of behavioral control processes is the post-error slowing (PES). PES reflects how an individual slows down the speed of their responses after they had made an error on a task. PES is commonly studied alongside ERN. Recent studies suggest that the magnitude of PES can be predicted by the neural correlates of the ERN signal (Chang et al., 2014; Fu et al., 2019). The relationship between ERN and PES has been found to persist throughout development and is associated with task accuracy (Ladouceur et al., 2007).

PES has been reported across neurodevelopmental and neurodegenerative conditions. For instance, in patients with PD, it has been used to understand medication and disease related modulation of cognitive control (Siegert et al., 2014). In ADHD, PES has been found to be consistently diminished across different developmental stages through to adulthood. It has been considered as a behavioral marker indicating ADHD symptomatology from an early childhood age (Balogh and Czobor, 2016). PES has not yet been investigated in neurodevelopmental motor disorders or in the context of motor ability in the general population.

### Control Processes and Anxiety

Whilst enhanced control processes on neural and behavioral levels may reflect a compensation that leads to better performance, this increased control may also have implications for the individual's emotional functioning, such as the experience of worries and anxiety.

Emotional difficulties are commonly observed in those with poor motor skills, especially in clinical populations such as DCD or TS, who report high levels of anxiety (Bloch and Leckman, 2009; Hill and Brown, 2013). However, a recent study reported that even healthy adults with relatively poorer motor proficiency experience higher levels of anxiety than those with better motor skills (Rigoli et al., 2017). The source of these emotional difficulties is yet to be investigated although, so far, it has been attributed to the environmental stress hypothesis (Cairney et al., 2010; Rigoli et al., 2017). The hypothesis was developed based on the experiences of children with DCD and suggests that motor difficulties expose individuals to negative experiences (such as not getting on with peers in school) that lead to negative self-perceptions and internalizing problems. Thus, the current explanation for anxious tendencies in relation to motor difficulties focuses on individual experiences and external factors whilst the possible impact of cognitive processes is yet to be considered.

The application of compensatory processes to support task performance could be another possible explanation for the observed high levels of anxiety in individuals with poor motor skills. It has been suggested that individuals who engage enhanced cognitive control efforts to facilitate their performance and avoid errors may be more sensitive to error making and they may be characterized by increased worries when committing errors (Frank et al., 2005; Holroyd and Umemoto, 2016). In fact, enhanced ERN is commonly observed in individuals with anxiety disorders (Weinberg et al., 2015), as well as undiagnosed individuals with high anxiety, which indicates that enhanced error control may be associated with trait anxiety (Hajcak et al., 2003). Investigating the anxious tendencies of individuals with poor motor functioning through this lens would help to understand the emotional risk factors that may be associated with their motor difficulties. Linking these risk factors with functioning at behavioral, cognitive and neural levels will be an important step forward in understanding the M-C interaction and its consequences, and it is to this end that the current study was conducted.

### The Current Study

The aim of the current study was to investigate the M-C interaction and its association with anxiety and worry in an adult general population with different motor proficiency profiles. By doing so, the study will elucidate whether the patterns and results seen in motor disorders are also present in the general population. Thus, the study evaluated two groups of healthy adults with higher motor proficiency (HMP group) and lower motor proficiency (LMP group). The patterns of cognitive and behavioral control mechanisms were measured through the ERN and PES during a standard Flanker task with congruent, incongruent and neutral conditions.

It was expected that both the LMP and HMP groups would show a typical flanker effect, i.e., accuracy would be lower and reaction times higher for the incongruent trials compared to congruent and neutral conditions (Hypothesis 1). In terms of cognitive control processes, it was predicted that the LMP group would have larger ERN amplitudes and longer PES than the HMP group (Hypothesis 2). Although PES has not yet been studied in association with motor functioning, this prediction was based on the reported association between the ERN and PES for efficient control of task performance. It was thus hypothesized that there would be a negative correlation between ERN amplitudes and PES across all participants, indicating that more negative error-related neural signals correspond to larger slowing of reaction times post-error (Hypothesis 3).

In terms of anxious tendencies, it was hypothesized that motor proficiency scores would correlate with anxiety scores across all participants (Hypothesis 4). Furthermore, it was expected that both ERN and PES would correlate with anxiety for the whole sample (Hypothesis 5). Specifically, it was expected that ERN would correlate with anxiety in the negative direction suggesting higher anxiety in relation to more negative ERN amplitudes. PES would correlate with anxiety in the positive direction, suggesting that larger slowing of reaction times corresponds to higher anxiety levels.

## Materials and Methods

### Participants

Participants were recruited by opportunity sampling either via the University of Surrey's research volunteer system or by word of mouth. First- and second-year psychology undergraduate students were offered two research tokens for participation which are required within their degree. Additional participants were postgraduate students or individuals working at the university. All participants were entered into a prize draw of two £50 shopping vouchers. Exclusion criteria comprised individuals below the age of 18 years old and/or those who had a diagnosis of neurodevelopmental, psychiatric or neurological disorders. A total of 40 participants were recruited. Demographic details are displayed in Table 1.

**Table 1:** Demographic information for participants in the overall sample and the two motor proficiency groups.

	**Overall sample**	**HMP**	**LMP**
*N*	40	23	17
Age in years	18–52 (*M* = 26.2, *SD* = 9.0)	18–52 (*M* = 28.0, *SD* = 9.7)	18–49 (*M* = 23.7, SD = 7.3)
Female participants (%)	60	52	76
Left-handed (%)	8	4	12
Student (BSc, MSc, PhD; %)	83	74	95
**Ethnic Background**			
White (%)	82.5	87	76
Black (%)	7.5	4	12
Asian (%)	7.5	9	6
Mixed (%)	2.5	0	6

### Materials

#### Motor Proficiency

The Bruininks–Oseretsky Test of Motor Proficiency 2nd Edition—short form; (BOT2-SF; Bruininks and Bruininks, 2005) was used to assess participants' motor proficiency. BOT2-SF is a standardized test with normative scores provided for ages 4–21. There are currently no standardized measures to test motor proficiency in adults over 21. BOT2-SF was chosen because its validity and reliability in participants up to the age of 21 is stronger in comparison to other available measures (Hands et al., 2015; McIntyre et al., 2017). BOT2-SF has previously been used in the study of adult populations (Sahlander et al., 2008; Du et al., 2015; Silva et al., 2017).

Motor proficiency is assessed in terms of four motor domains: fine manual control, manual coordination, body coordination, and strength and agility. In the strength and agility domain, the researchers make a choice with regards to the type of push-up participants complete as part of the test. In this study, knee push-ups were chosen. The raw scores from the assessment were totaled and used in the analyses. Higher scores indicate better motor proficiency.

A median split method was used to allocate participants into two groups with higher or lower motor proficiency scores. The final scores ranged from 62 to 79 with the mean of 71.48 and the median of 72. Therefore, 23 participants with a score of 72 or more were placed in a high motor proficiency (HMP) group and 17 participants with a total score of 71 or less were placed in the lower motor proficiency group (LMP). In this group, six participants' motor proficiency scores fell below the 10th percentile indicating clinically significant motor difficulties and a possibility of undiagnosed DCD. The percentile calculations were based on age-appropriate scale scores up to the age of 21. As no more scale scores are available beyond this age, the remaining calculations were based on the scale scores for 21-year olds. Some participants' raw scores equated to the mean/median score and so the resulting two groups were uneven. It has been suggested that, in such situations, the power of the study may be reduced (Iacobucci et al., 2015). However, the study included group analyses as well as correlational analyses and thus it was important to include individuals representing the whole range of motor skills in order to test linear relationships between the investigated variables.

#### Cognitive Measures

The Flanker task (Eriksen and Eriksen, 1974) was presented during an EEG recording using E-Prime 2.0 software (Psychology Software Tools, 2012). Each trial consisted of seven arrowheads presented in Arial 24 pt at the center of the screen. The middle arrowhead was the target. Participants were instructed to press the letter “C” if the middle arrowhead was pointing left and the letter “M” if the arrowhead was pointing right on a standard computer keyboard. The three arrowheads positioned on each side of the target were distractors. The orientation of the distractor arrowheads was dependent on the test condition. In the congruent condition, the distractor arrowheads were pointing in the same direction as the target and in the opposite direction in the incongruent condition. A neutral condition was also included where distractor arrowheads were pointing down, resembling the letter “V.” Each trial was preceded by a fixation cross.

**Figure 1:**
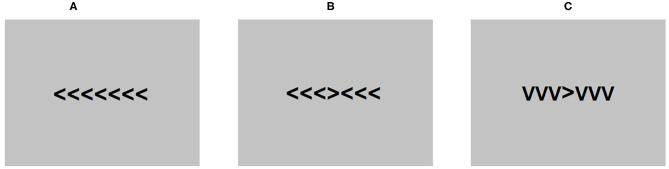
An example of the conditions presented in the flanker task. **(A)** is the congruent condition, **(B)** is the incongruent conditions, and **(C)** is the neutral condition.

The task was composed of 600 trials, including 200 trials in each condition, pseudorandomized across four blocks of 150 trials. Participants were instructed to take a break between each block and rest their eyes to avoid excessive blinking throughout the procedure. The time allowed to make a response in each trial was 600 ms and between-trial intervals were jittered between 400 and 1,600 ms to prevent rhythmical responses. Figure 1 shows a diagram of the task conditions as presented to the participants.

Apart from task accuracy and reaction times, PES was extracted using the robust method proposed by Dutilh et al. (2012b). The method was chosen because it produces more reliable results in cases where reaction time variability may fluctuate throughout the task. It was expected that this could be the case in a Flanker task with three conditions of different difficulty and participants were expected to respond more slowly in the incongruent compared to the congruent condition. Before the PES was calculated, all omission trials were removed from the analysis because they differ from the commission errors in the pattern of response and may lead to post-omission speeding (Huang et al., 2013). PES was calculated in a pairwise approach around each commission error using the following formula (Dutilh et al., 2012b):

(1) $$\begin{array}{r} PES=\;\frac{1}{n}\;\sum_{i\;=\;1}^{n}\left(RT\left[E_{i+1}\right]-RT\left[E_{i-1}\right]\right) \end{array}$$

Here, *n* is the total number of commissioned errors, *E*_*i*_ is the *i*th commission error trial, *RT*[*E*_*i*__−1_] and *RT*[*E*_*i*__+1_] are reaction times of the preceding and the subsequent correct trial, respectively. It was also required that *E*_*i*__−2_ was a correct response because otherwise *E*_*i*__−1_ (included in the formula) would have been a post-error reaction time (RT) and could bias the calculation.

A total of 11 participants were excluded from all PES analyses, resulting in the following group sizes, HMP *N* = 17, LMP *N* = 12. Ten of these cases were removed because participants committed fewer than 11 errors which were suitable for PES analysis, as calculated with the formula presented above. Reaction time data tends to be very variable and it is suggested that as many trials as possible should be retained to obtain a representative mean for each participant. Danielmeier and Ullsperger (2011) suggest that experimenters should exercise caution, but so far there is no recommendation for the minimal number of erroneous trials that should be included in PES analyses. In the current experiment, it was identified that 25% of participants committed fewer than 11 errors qualifying for the analysis of PES. It was not feasible to raise the threshold of 11 trials any higher as this would lead to many more exclusions and the current analysis would not be sensitive enough to identify the effects of interest. One additional case was removed which was an extreme outlier with a large number of committed errors (*z* =2.39). The mean of qualifying PES errors did not significantly differ between the two groups when tested with an independent samples *t*-test (HMP *M* = 24.18, LMP *M* = 27.54, *t* = 0.24, *p* = 0.467, *d* = 0.27).

#### Neurophysiological Measures

The aim of the EEG measurement was to obtain the error-related negativity (ERN) signal. EEG data were acquired using the BrainCap (Neurospec) with 32 Ag/AgCl sintered electrodes in an extended 10/20 system. Impedance was kept below 5 kΩ. Vertical and horizontal eye movements were recorded by electrodes located below and on the side of the left eye. Linked mastoid electrodes were used as the online reference and the ground electrode was located at the AFz location. Data were digitized at a sampling rate of at 500 Hz. There was a high cut-off online filter implemented at 250 Hz. Offline analysis was carried out using BrainVision Analyzer 2 (Brain Products, 2012). A bandpass filter 0.5–40 Hz was applied to the raw data. Eye movement correction was applied using the automatic independent component analysis approach for ocular correction following the default values across the whole dataset. Segments were sourced separately for correct and incorrect responses across all conditions within −150 and 200 ms of the response. Additional artifacts were rejected following the default values for the gradient, difference of values in intervals, minimal and maximal amplitude, and low activity. Baseline correction was applied with baseline period at 150–100 ms prior to the response before the respective segments were averaged.

Only participants with 8 or more artifact free error trials were included for ERN analyses, given previous suggestions that a minimum of 6–8 trials should be included for good internal consistency (Olvet and Hajcak, 2009). This criterion resulted in the exclusion of two participants in the HMP group from all analyses involving ERN. The error count in the retained sample ranged from 11 to 73 with the mean of 33.18 across the whole sample and no significant difference between the two groups [HMP *M* = 31.19, LMP *M* = 35.65, *t*_(36)_ = 0.82, *p* = 0.419, *d* = 0.27]. Only recordings from the Cz channel were used to measure the ERN. ERN mean amplitudes were extracted for a 50 ms time window from 10 to 60 ms post-response separately for correct and incorrect responses. The respective trials were pooled together from the three conditions—congruent, incongruent, and neutral—as most participants did not commit enough errors to allow for a comparison between conditions. Similar as with PES, all omission trials were excluded as omission-related brain activity has been reported to be different to commission-error related activity and primarily linked to attentional processes (Perri et al., 2017; Yokota et al., 2019).

A difference wave was calculated to represent the ERN by subtracting the observed amplitude on the trials with correct responses from those with incorrect responses. This is an efficient method that has been shown to reduce the overlap with other event related potentials (Luck, 2014) and is more robust in comparison to other available techniques, such as only using the mean amplitude following incorrect responses or the difference between the post-error negativity and the preceding positivity (Luck, 2014; Fischer et al., 2017).

#### Trait Anxiety

The Penn State Worry Questionnaire (PSWQ; Meyer et al., 1990) was used to measure the levels of trait worry to reflect participants' trait anxiety tendencies. The scale has been validated to be a suitable tool for the investigation of the nature of worries as a trait (Olatunji et al., 2007). PSWQ comprises 16 items which are answered on a 5-point Likert scale where 1 indicates “not at all typical of me” and 5 indicates “very typical of me.” A maximum score on the questionnaire is 80. Ascending scores reflect higher anxiety and a score of 60 or above reflects high levels of worry compared to normative samples (Gillis et al., 1995). PSWQ has been shown to successfully distinguish participants with a generalized anxiety disorder who experience excessive worry from other forms of anxiety such as social anxiety or obsessive-compulsive disorder (Brown et al., 1992; Fresco et al., 2003). Thus, it can be used as a measure of anxiety reflecting traits of excessive worry (Fresco et al., 2003). The PSWQ has previously been used in the study of the association between anxiety and error-related cognitive control in the general population (Hajcak et al., 2003; Pajkossy et al., 2017).

### Procedure

Ethical approval was obtained from the Faculty of Health and Medical Sciences Ethics Committee at the University of Surrey. Participants received the study information sheet when first expressing interest in the study and then again before signing the consent form when arriving in the room set up for the procedure. All participants were aware of the nature of the study. Namely, they were told that the relationship between their motor skills, anxiety/worry and cognitive control processes would be studied. After signing the consent form, they were asked to complete the demographic and PSWQ questionnaires. Then, they were taken to an adjacent room where they completed the BOT2-SF tasks. Finally, they were taken to a smaller EEG room where the EEG cap was applied. For each participant, the scalp was cleaned with an alcohol solution where the electrodes were placed, and an electrolyte paste was used for conductance. Once the setup was finished, participants completed the Flanker task. On average, the duration of the whole session from participant arrival to departure was 1 h and 45 min including breaks. The whole procedure was conducted in the same order by the same researcher for each participant. Participants were debriefed after the study to further clarify that the EEG analysis would focus on the instances when they made errors during the task.

### Data Analysis

Statistical analysis of the data was conducted using SPSS 24 (IBM Cor, 2016). All variables used to test the hypotheses were tested for normality in their distribution. Age and PES were log-transformed due to significant skew and kurtosis. A screening process included a correlational investigation of the likely effects of sample characteristics (age and gender) on the dependent variables including ERN, PES, and anxiety and motor functioning. There was a significant positive correlation between age and motor proficiency (*r* = 0.435, *p* = 0.005).

For the analysis of the flanker effects, the whole sample of 40 participants was included (HMP *N* = 23, LMP *N* = 17). The behavioral results included reaction times (RT), with omission trials excluded, and accuracy (proportion of correct responses), where omission trials were retained. This was collected for both groups across the three conditions. The differences between flanker conditions (congruent, incongruent and neutral) and motor proficiency groups (HMP and LMP) were tested using a two-way mixed ANOVA.

For the investigation of performance control processes, the ERN difference wave mean amplitudes were compared between the two motor proficiency groups (HMP *N* = 21, LMP *N* = 17) at the Cz channel using a two-tailed independent samples *t*-test. A two-tailed independent samples *t*-test was used to test the difference between the mean PES scores for both groups (HMP *N* = 17, LMP *N* = 12). The relationship between ERN and PES was measured using a Pearson correlation.

For the correlational analyses involving anxiety, a one-tailed partial correlation, controlling for age, was conducted to test the relationship between motor skills and anxiety. In this analysis, one multivariate outlier was removed, and the total of included cases was *N* = 39. Age was included as a controlled variable because a significant relationship with motor skills had been identified during the screening of the variables. Subsequently, one-tailed partial correlations, controlling for motor skills, were conducted to test the relationship between anxiety and ERN as well as anxiety and PES. Motor proficiency was partialled out due to the expected relationship between motor skills and anxiety.

### Exploratory Data Analysis

Further analyses were informed by the outcomes from the hypothesis-driven analyses. These focused on participants' accuracy and the type of errors they made to investigate whether cognitive control and behavioral adaptation helped participants to perform accurately and avoid errors. Correlational analyses were used to test the relationship between ERN/PES and accuracy. Additionally, the total number of errors per each participant was divided into two new variables, total omissions and total commissions. The variables were normally distributed and thus the differences between the total number of omissions and commissions were compared for both motor proficiency groups using independent *t*-test analyses. This was decided because omission errors could be associated with PES due to the slowing of reaction times and a consequent inability to execute the responses in time. The analyses were Bonferroni corrected.

## Results

### Flanker Effect

Accuracy means and standard deviations across task conditions are displayed in Table 2 for each of the two groups. A two-way (flanker condition, motor proficiency group) mixed ANOVA with applied Greenhouse–Geisser correction revealed a significant effect of condition, *F*_(1.14, 76)_ = 100.40, *p* < 0.001, η_*p*_^2^ = 0.73, η_*G*_^2^ = 0.53. The effect of motor proficiency group was also significant, *F*_(1, 38)_ = 6.77, *p* = 0.013, η_*p*_^2^ = 0.15, η_*G*_^2^ = 0.09 with the LMP group scoring below the HMP group overall. There was no significant interaction between group and condition, *F*_(1.14, 76)_ = 1.31, *p* = 0.264, η_*p*_^2^ = 0.03, η_*G*_^2^ = 0.01. This indicates that there was a significant general effect of the flanker condition on accuracy which is further confirmed by *post-hoc t*-test comparisons for all condition pairs. The results are presented in Table 3.

**Table 2:** Mean and standard deviations for accuracy proportion and reaction times (ms) on the flanker task for each condition per motor proficiency group.

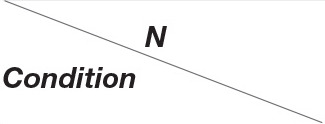	**HMP**	**LMP**
	**23**	**17**
**Accuracy**		
Congruent	0.93 (0.07)	0.88 (0.09)
Neutral	0.71 (0.15)	0.61 (0.14)
Incongruent	0.92 (0.07)	0.86 (0.09)
**Reaction Time**		
Congruent	432 (33.5)	448 (32.3)
Neutral	440 (31.4)	452 (28.9)
Incongruent	483 (26.2)	488 (27.4)

**Table 3:** *T*-test *post-hoc* comparisons reflecting the flanker effect.

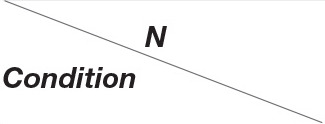	**HMP**	**LMP**
	**23**	**17**
**Accuracy**		
Congruent vs. Neutral	0.30 (1.0)	0.85 (1.0)
Congruent vs. Incongruent	8.51 (<0.001)	9.40 (<0.001)
Incongruent vs. Neutral	8.25 (<0.001)	8.538 (<0.001)
**Reaction Time**		
Congruent vs. Neutral	−2.08 (0.617)	−1.03 (1.0)
Congruent vs. Incongruent	−13.35 (<0.001)	−9.15 (<0.001)
Incongruent vs. Neutral	−11.28 (<0.001)	−8.12 (<0.001)

*T-value (p-value) results are displayed for all condition pairs across the HMP and LMP groups. Bonferroni correction was applied to all p-values*.

Reaction times means and standard deviations across task conditions are also displayed in Table 2. A two-way (flanker condition, motor proficiency group) mixed ANOVA with applied Greenhouse–Geisser correction also revealed a significant effect of condition for RTs, *F*_(1.21, 76)_ = 143.9, *p* < 0.001, η_*p*_^2^ = 0.79, η_*G*_^2^ = 0.31. However, the group effect was not significant, *F*_(1, 38)_ = 1.44, *p* = 0.237, η_*p*_^2^ = 0.037, $\eta_{G}^{2}$ = 0.03 and neither was the interaction *F*_(1.21, 76)_ = 1,628, *p* = 0.203, η_*p*_^2^ = 0.04, η_*G*_^2^ = 0.005. This indicates that there was a significant general effect of the flanker condition on RTs which did not differ between the two groups. *Post-hoc t*-test comparisons of RTs for condition pairs across each group are presented in Table 3.

### Cognitive Control and Behavioral Adaptation

**Figure 2:**
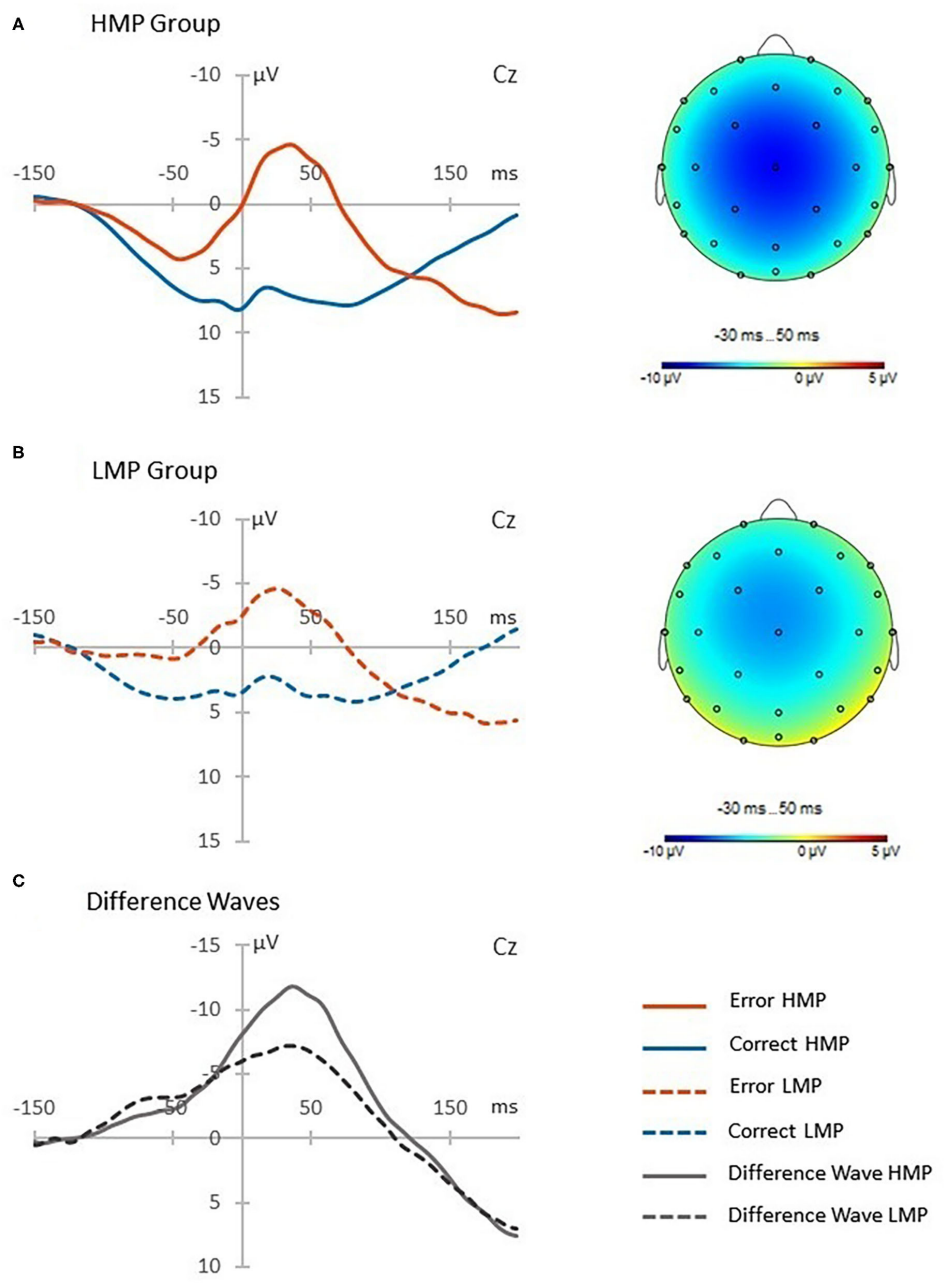
Post-error and post-correct waveforms presented for each motor proficiency group. The high motor proficiency group is presented in panel **(A)** and the low motor proficiency group is presented in panel **(B)**. The difference waves in panel **(C)** reflect the error-related negativity (ERN) response which is additionally presented with a topographic distribution for each group.

The difference in the ERN (μV) between HMP (*N* = 21, *M* = −11.45, *SD* = 7.63) and LMP (*N* = 17, *M* = −6.76, *SD* = 5.24) groups at the Cz channel was tested using a two-tailed *t*-test with Welch correction. The HMP group had significantly higher ERN *t*_(35)_ = 2.24, *p* = 0.031, *d* = 0.71 than the LMP group. Figure 2 shows the obtained post-error, post-correct and difference waves at the Cz channel as well as the topographic distributions of the difference waves for both groups.

**Figure 3:**
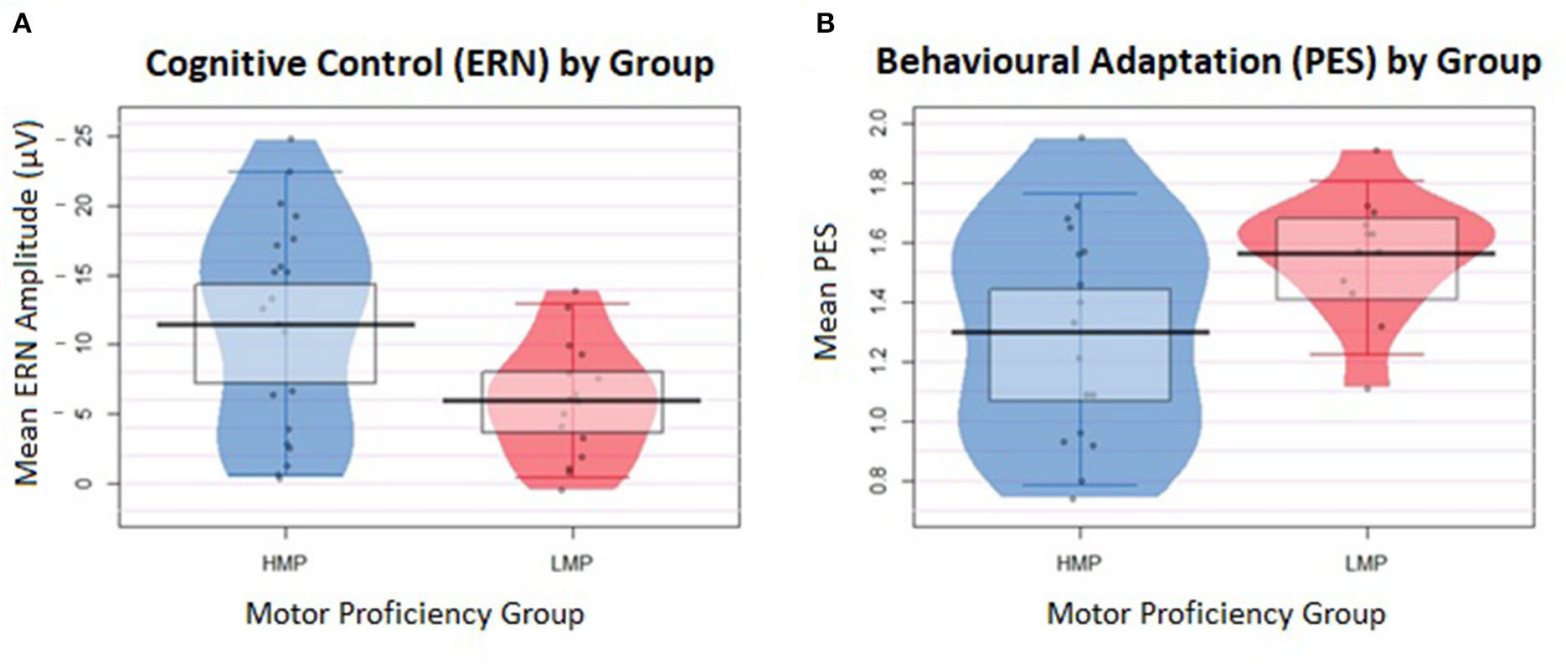
Individual data are displayed in the form of jittered dots and data density, indicated by the width of the shape. The mean value is marked by the black indicator line and the box around it marks the high-density intervals of the mean. **(A)** Differences in the error-related negativity (ERN) values between the two groups. ERN values were inverted from negative to positive values to represent larger ERN at the top of the plot and smaller ERN at the bottom. **(B)** Differences in the post-error slowing (PES) values between the two groups.

Additionally, a two-tailed *t*-test with Welch correction was conducted on the log-transformed PES values between the HMP (*N* = 17, *M* = 1.30, *SD* = 0.36) and LMP (*N* = 12, *M* = 1.56, *SD* = 0.21) groups. The LMP group had significantly longer PES than the HMP group, *t*_(26.19)_ = 1.40, *p* = 0.021, *d* = 0.88. Figure 3 shows a raw descriptive-inferential graph for the group differences on ERN and PES.

Contrary to Hypothesis 3, the correlation between the ERN and PES was not significant, *N* = 29, *r* = −0.041*, p* = 0.833.

### Cognitive Control, Behavioral Adaptation, and Anxiety

**Figure 4:**
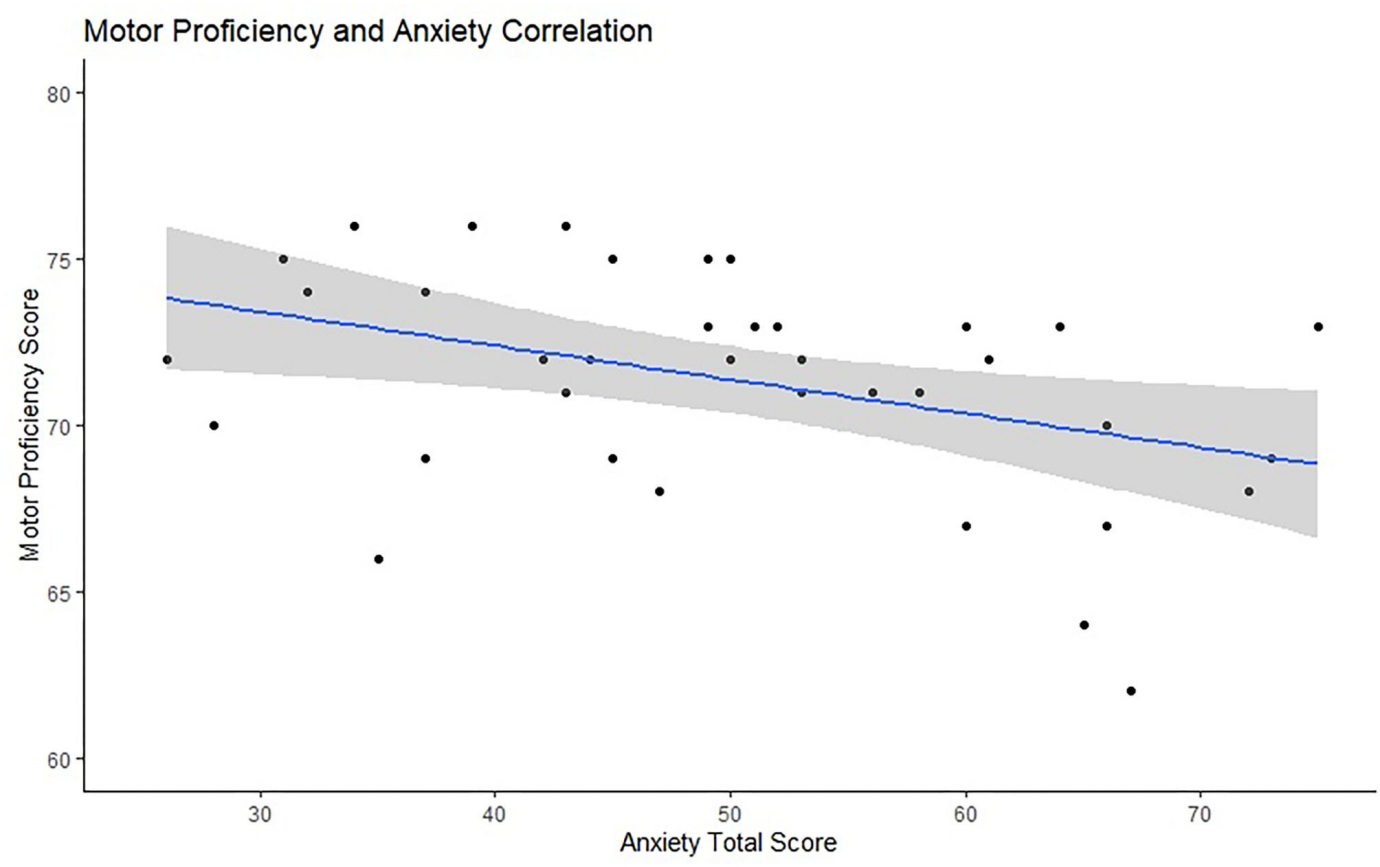
Visual representation of the relationship between anxiety scores (*x*-axis) and motor proficiency (*y*-axis) with a marked line of best fit and the confidence intervals reflected with the shadowed area.

The partial correlation between anxiety and motor proficiency, controlled for participant age, was significant, *r* = –0.360, *p* = 0.027. This suggests that anxiety is associated with participants' motor proficiency. Figure 4 presents the scatter plot for this relationship. The partial correlation between ERN and trait anxiety, controlled for motor skills, was not significant, *N* = 38, *r* = 0.19, *p* = 0.13. Similarly, the partial correlation between PES and trait anxiety was also not significant, *N* = 29, *r* = 0.251, *p* = 0.198.

### Exploratory Analyses

**Figure 5:**
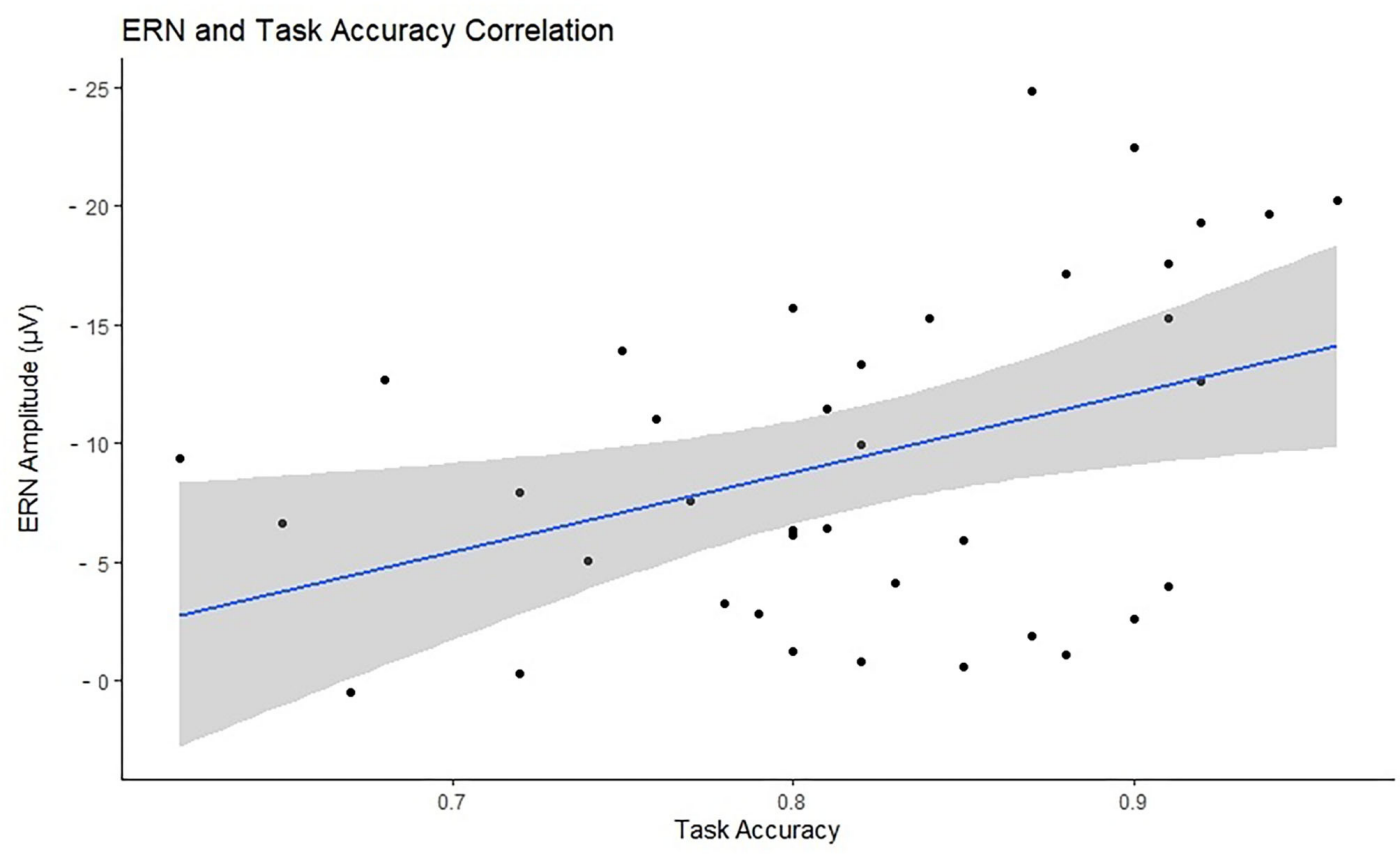
Visual representation of the relationship between task accuracy (*x*-axis) and the error-related negativity (ERN) amplitudes (*y*-axis) with a marked line of best fit and the confidence intervals reflected with the shadowed area. The ERN values have been reversed for visualization purposes as the more negative the ERN the stronger the cognitive control signal.

Accuracy was tested in relation to ERN and PES to investigate the efficiency of cognitive control and behavioral adaptation in individuals with different levels of motor proficiency. Accuracy significantly correlated with ERN in the negative direction (*N* = 38, *r* = –0.398, *p* = 0.026), indicating that higher accuracy is associated with larger ERN. There was no significant correlation between accuracy and PES (*N* = 29, *r* = −0.051, *p* = 0.791). Figure 5 presents the scatterplot for the relationship between ERN and accuracy. The total number of omission and commission errors was extracted for both motor proficiency groups; the descriptive statistics are provided in Table 4. The LMP group made significantly more omissions compared to the HMP group [*t*_(38)_ = 2.40, *p* = 0.044, *d* = 0.77]. There was no difference for commission rates [*t*_(38)_ = 1.21, *p* = 0.466, *d* = 0.39].

**Table 4:** Means and standard deviations for the total number of omission and commission errors for the two motor proficiency groups.

	**HMP**	**LMP**
Omission Errors	59.74 (40.8)	93.65 (48.7)
Commission Errors	29.00 (16.4)	35.65 (18.2)

## Discussion

The current study aimed to investigate the motor-cognitive interaction in the general population by focusing on cognitive control and behavioral adaptation in individuals with different levels of motor proficiency. It also investigated whether these mechanisms were associated with anxiety.

### Cognitive Control and Behavioral Adaptation

First, in order to assess the cognitive control and behavioral adaptation mechanisms, healthy adult participants were split into two groups based on their motor ability including high motor proficiency (HMP) and low motor proficiency (LMP). They completed the flanker task during an EEG recording. Both groups demonstrated longer reaction times and lower accuracy in the incongruent condition of the task as expected. This has been regularly reported in previous studies and indicates that the task is completed correctly, justifying further analyses (Grützmann et al., 2014; Fischer et al., 2016).

#### Error-Related Negativity

For the purpose of this study, cognitive control was indexed by the ERN signal. It was found that the LMP group had significantly lower mean ERN compared to the HMP group. This suggests that individuals with poorer motor proficiency may have attenuated activity in the ACC or more varied latency of the error-related neural response in comparison to individuals with better motor proficiency. This contradicts the claims by Querne et al. (2008) and Schüller et al. (2018), who suggest that ACC activity may be enhanced in individuals with motor control difficulties in order to facilitate task accuracy in a compensatory manner.

One explanation for the contradicting results could be that individuals with DCD or TS are more likely to develop cognitive mechanisms to facilitate their performance despite experienced difficulties. Both DCD and TS are neurodevelopmental disorders affecting individuals since childhood, thus giving an opportunity to seek professional help and over the years develop management strategies for a range of different situations and activities. This could lead to the optimization of cognitive control and a different pattern of activity within the ACC for those individuals. In comparison, individuals who have no motor disorders but present with poorer motor functioning may not see their motor performance to be problematic and they may not receive specialist input to help with the development of compensatory strategies.

On the other hand, the M-C interaction may be syndrome-specific and operate differently for different types of motor disorders, which may explain why results from the general population do not reflect those from studies on motor disorders.

#### Post-error Slowing

Behavioral adaptation was indexed by the reaction-time based PES. As expected, it was found that the LMP group had significantly larger PES compared to the HMP group. Since motor proficiency in this group was below average, it is inferred that the task was more challenging for these individuals. Longer PES in this group may also reflect that the task required more effort. In order to maintain good performance, participants were more careful and adapted their responses when making errors. PES is a self-regulatory behavioral control mechanism characteristic of individuals who obtain high task accuracy (Steinborn et al., 2012). Thus, it can be interpreted as a compensatory behavioral mechanism for this group.

Contrary to previous research on ERN and PES, the current study found no evidence for a relationship between these two performance control mechanisms.

### Task Accuracy

There was an unexpected significant effect of group on task accuracy wherein the LMP group performed worse than the HMP group. The finding is unsurprising because the LMP group was likely to find the task more challenging. However, it is also unexpected because previous research found that participants with motor disorders including DCD and TS completed reaction-time-based cognitive tasks with accuracy rates that were not different from those of control groups (Querne et al., 2008; Schüller et al., 2018). This led to the assumption that cognitive control and behavioral adaptation must help individuals with motor disorders to compensate for their motor difficulties and perform the tasks as well as healthy individuals.

It should be noted that although both Querne et al. (2008) and Schüller et al. (2018) observed no statistical significance in the difference between the accuracy of the clinical and control groups, without equivalence testing or Bayesian analysis this is not evidence for equivalent task performance. Therefore, it does not preclude that there might be differences in other cohorts. The current study suggests that task accuracy may differ between generally healthy individuals with better and worse motor ability.

#### Exploratory Analyses

The matter of task accuracy is an important point in research on performance control as it directs the interpretation of findings reflecting cognitive control and behavioral adaptation mechanisms. As a result of the exploratory analyses, a significant medium-large correlation between task accuracy and ERN was found, but the correlation between PES and task accuracy was not significant. This suggests that ACC cognitive control processes, as reflected by the ERN, facilitate successful task performance. This effect has been shown in previous research (Themanson et al., 2012) and is consistent with the models of cognitive control and the proposed patterns of interaction between motor and cognitive networks within the ACC (Holroyd and Coles, 2002; Brown and Alexander, 2017). It is therefore possible that the HMP group benefitted from strong cognitive control processes and thus achieved better task accuracy compared to the LMP group.

On the other hand, the study did not find evidence that behavioral adaptation in the form of PES may contribute to the improvement of task accuracy, given that the tested correlation produced a very negligible effect size of *r* = −0.051, which was not statistically significant. It may be the case that the extended reaction times occurring after error commission have led participants in this group to make omission errors. They slowed their RTs to the extent where they were not able to execute their prepared response in time before the next trial began. To explore this possibility, the rate of commission and omission errors was compared between the two groups and it was found that the LMP group made significantly more omissions but not commissions compared to the HMP group. This suggests that their application of PES might have been counterproductive and could have contributed to the difference in task accuracy between the two groups. Perhaps participants in the LMP group were likely to prioritize accuracy over speed, considering omission errors as less indicative of their ability to perform the task correctly. These suggestions are however speculative and should be confirmed with further research.

### Anxiety and Performance Control

Cognitive control (ERN) and behavioral adaptation (PES) were both tested for their relationship with trait anxiety following previous reports linking enhanced control mechanisms with increased anxiety levels (Hajcak et al., 2003). There was a significant relationship between motor proficiency and anxiety in line with previous research (Rigoli et al., 2017) thus providing a good opportunity to investigate whether this could be associated with participants' sensitivity to error making and the need to apply cognitive control processes and behavioral adaptation mechanisms.

There was no significant correlation between ERN or PES with trait anxiety. For PES, the account of this relationship has been inconsistent in previous reports. It has been shown that anxious individuals tend to slow down on tasks when making errors (Dutilh et al., 2012a) but also that anxiety does not interact with PES and instead impacts post-error accuracy (van der Borght et al., 2016). For the present study, the application of behavioral control mechanism in the form of PES cannot be associated with increased levels of anxiety in individuals with lower motor proficiency.

Previous reports concerning clinical populations, suggest a strong link between ERN and anxiety (Holroyd and Umemoto, 2016, Weinberg et al., 2015). This effect may not be observable in the current study composed of generally healthy and subclinical samples. Especially due to the fact, that the group which was more likely to experience high levels of anxiety was characterized by attenuated ERN. Further research is needed to understand whether anxiety in relation to motor difficulties is mainly due to environmental factors as suggested by the environmental stress hypothesis (Rigoli et al., 2017) or whether it could be in part due to the effort needed to apply compensatory behavioral or cognitive mechanisms.

### Implications

From the results reported above, it occurs that individuals with different motor proficiency levels may be characterized with qualitatively different performance control profiles. The HMP group, with better motor proficiency levels, appears to control their performance with more automatic and effortless mechanisms. They had higher neural activity in response to errors which was associated with their high task accuracy.

On the other hand, the LMP group, with poorer motor proficiency, attempted to control their performance with a more strategic and effortful approach. They showed larger PES which can be interpreted as an attempt to be more careful in their responses. However, because this approach increased the chances of omission errors, their accuracy rate was reduced. This reflects that their performance control approach may not be efficient in all situations. The Flanker task is a fast-paced task which doesn't leave much room for slowing down before the trial is omitted. Perhaps the application of PES could be more efficient in situations where taking more time to respond or complete the task does not lead to repercussions. This line of research could be investigated with the use of tasks which resemble everyday activities to learn about the use of behavioral adaptation and slowing of responses by individuals with lower motor proficiency in more ecologically valid settings. This could subsequently inform new interventional solutions for support in educational and occupational settings.

In the context of the M-C interaction, the differing performance control profiles suggest that lower motor proficiency may be related to reduced cognitive control resources and as a result, the correction of erroneous behaviors requires more effort. This can be considered in the context of motor rehabilitation in individuals with poor motor functioning including those with intellectual disability. Motor difficulties are often observed in such groups (Cantone et al., 2018; Jeoung, 2018). Furthermore, it has been reported that motor rehabilitation helps to improve motor functioning in individuals with intellectual disability and this has been linked to the mechanisms of neural plasticity (Cantone et al., 2018). Therefore, it should be investigated whether motor rehabilitation may lead to changes in the ACC activity and facilitate a more automatic mechanism for performance control as observed in the HMP group of the current study. This would be a major advantage for individuals with lower motor proficiency as the control over task performance could potentially become more accurate and less effortful.

The significant relationship between motor skills and anxiety should be considered by health professionals and in the context of educational and occupational settings. This could help to ensure that adequate support is provided to individuals with significant motor difficulties in terms of the associated emotional impact which may be less obvious than the motor difficulties themselves.

### Limitations

The main limitation of the current study is the use of the median split method to create the two groups with different motor proficiency levels. A regression approach was not suitable as the aim of the study was to understand the characteristics of cognitive and behavioral control processes in individuals with different motor proficiency profiles. Participants who were recruited reflected a wide range of motor skills with many cases nesting around the median score of 72 on the motor proficiency assessment, which made the two resulting groups uneven in size. This could have affected the power of the study and made it less likely to detect significant differences.

However, the study also tested linear relationships between the observed variables and therefore it was important to recruit participants whose motor proficiency scores would form a continuous variable. In addition, the two resulting groups were significantly different on their motor proficiency level, with a large effect size of more than 2 standard deviations (*d* = 2.58). In Figure 3, it is evident that the median split worked well to separate a homogenous group of participants with lower motor proficiency (LMP), which may be seen as a sub-clinical group with consistently small variance across their ERN and PES. In comparison, the HMP group was more heterogenous, with individuals often obtaining ERN or PES comparable to that of participants in the LMP group, which could be for reasons other than their motor proficiency. In general, the current study obtained a sufficient sample to detect the effects of interest despite the uneven split.

One additional disadvantage of the study is the possibility of sampling bias. A significant positive correlation between age and motor proficiency was found, suggesting that the older the participants the more likely they were to present with better motor skills. It is widely understood that motor skills decline as a function of age in the general population (Leversen et al., 2012). It is possible that older individuals with relatively better motor skills than their peers were more likely to volunteer and take part in the study as they felt more confident about their performance. In the current study, all ANOVA and correlational analyses on motor proficiency were controlled for the effect of age. However, future research may aim to plan their recruitment strategy to be equally inviting for those who have different levels of motor proficiency across ages to avoid sampling biases.

Lastly, there are comparisons drawn between the results of this study and other studies conducted with participants with DCD and TS (Querne et al., 2008; Schüller et al., 2018). The comparisons are only relative considering that the methodology used in the current study was different. To support the conclusions of this study and the understanding of the M-C interaction in the context of healthy individuals as well as those with motor disorders, future research, and any replications of the current project should aim to make direct comparisons between clinical and sub-clinical groups using the same methodology.

## Conclusion

The lower motor proficiency group engaged cognitive control processes less efficiently than the individuals in the high motor proficiency group. Instead, they adapted their task performance behaviorally through slowing their reaction times, which was less effective. This suggests that the interaction between motor and cognitive processes in healthy individuals may not be comparable to that of individuals with motor disorders. The results of this study provide an important baseline for the understanding of the changes in motor-cognitive processes in clinical, sub-clinical, and healthy populations.

## Data Availability Statement

The datasets presented in this article are not readily available due to ethical constraints. Participants in this study did not give consent for their data to be made publicly available. However, the data may be requested for further research subject to relevant legal, professional, and ethical approval. Questions about the dataset and its availability can be directed to the corresponding author MT by email at m.topor@surrey.ac.uk.

## Ethics Statement

The study procedures involving human participants have been reviewed against the guidelines set out by the Ethics Committee of Faculty of Health and Medical Sciences, University of Surrey and carried out in accordance with the University of Surrey's Code of Conduct on Good Research Practice. Participants provided their written informed consent to participate in this study.

## Author Contributions

The following contributions are specified following the CRediT taxonomy. MT: conceptualization, data curation, formal analysis, investigation, methodology, project administration, validation, visualization, writing – original draft. BO: methodology, formal analysis, software, supervision, validation, visualization, writing – review and editing. HL: conceptualization, supervision, writing – review and editing. All authors contributed to the article and approved the submitted version.

## Conflict of Interest

The authors declare that the research was conducted in the absence of any commercial or financial relationships that could be construed as a potential conflict of interest.

## Abbreviations

ACC: Anterior cingulate cortex
ADHD: Attention deficit hyperactivity disorder
BOT2-SF, Bruininks–Oseretsky test of motor proficiency, 2nd edition: short form
EEG: Electroencephalography
ERN: Error-related negativity
DCD: Developmental coordination disorder
fMRI: Functional magnetic resonance imaging
HMP: High motor proficiency group
LMP: Low motor proficiency group
M-C Interaction: Motor-cognitive interaction
PES: Post-error slowing
PD: Parkinson's Disease
PSWQ: Penn State Worry Questionnaire
RT: Reaction time
TS: Tourette's Syndrome.
